# Population relationships based on 170 ancestry SNPs from the combined Kidd and Seldin panels

**DOI:** 10.1038/s41598-019-55175-x

**Published:** 2019-12-11

**Authors:** Andrew J. Pakstis, William C. Speed, Usha Soundararajan, Haseena Rajeevan, Judith R. Kidd, Hui Li, Kenneth K. Kidd

**Affiliations:** 1 Department of Genetics, Yale University School of Medicine, New Haven, CT 06520 USA; 2 Center for Medical Informatics, Yale University School of Medicine, New Haven, CT 06520 USA; 3 Ministry of Education Key Laboratory of Contemporary Anthropology, School of Life Sciences, Fudan University, Shanghai, 200433 China

**Keywords:** Genetic markers, Haplotypes

## Abstract

The benefits of ancestry informative SNP (AISNP) panels can best accrue and be properly evaluated only as sufficient reference population data become readily accessible. Ideally the set of reference populations should approximate the genetic diversity of human populations worldwide. The Kidd and Seldin AISNP sets are two panels that have separately accumulated thus far the largest and most diverse collections of data on human reference populations from the major continental regions. A recent tally in the ALFRED allele frequency database finds 164 reference populations available for all the 55 Kidd AISNPs and 132 reference populations for all the 128 Seldin AISNPs. Although much more of the genetic diversity in human populations around the world still needs to be documented, 81 populations have genotype data available for all 170 AISNPs in the union of the Kidd and Seldin panels. In this report we examine admixture and principal component analyses on these 81 worldwide populations and some regional subsets of these reference populations to determine how well the combined panel illuminates population relationships. Analyses of this dataset that focused on Native American populations revealed very strong cluster patterns associated with many of the individual populations studied.

## Introduction

DNA-based polymorphisms that differ substantially in frequency among human populations can be employed to infer ancestry. The potential utility of ancestry informative markers (AIMs), especially ancestry informative SNPs (AISNPS), for forensic, anthropological, and medical applications has been reflected in part by a large number of research reports. Soundararajan *et al*.^1^ showed that the 21 AIM panels published to that time had very little overlap of SNPs. More panels have been published in the intervening years and the minimal overlap in SNPs in the various panels remains a problem. Many of the studies in the literature involve a unique set of SNPs evaluated on a unique set of population samples. The 1000 Genomes data have been analyzed for several sets of AIMs but those populations do not represent an ideal forensic sample of human diversity around the globe because they do not include the full range of genetic diversity. Reviews dealing with aspects of AIMs such as human identification, ancestry inference, mixture deconvolution, and panels for predicting individual phenotypes for eye, hair, and skin color have appeared^1–7^. The benefits of AISNPs have not been realized as quickly as they might have for various reasons. Multiple factors have combined to make it difficult to evaluate and compare the general utility of most of the proposed AISNP sets. The benefits of particular AISNP and other AIM panels can only accrue and be properly evaluated as the data for them accumulate and become readily accessible on a large number of reference populations approximating the genetic diversity of human populations worldwide.

A recent tally from the ALlele FREquency Database (ALFRED: https//alfred.med.yale.edu) and the Forensic Reference-Resource on Genetics knowledge base (FROG-kb: https//frog.med.yale.edu) shows that complete frequency data have accumulated on 132 reference populations for the 128 Seldin AISNPs and on 164 reference populations for the 55 Kidd AISNPs. The union of the two panels encompasses 170 different autosomal AISNPs; the panels have 13 SNPs in common. Subsets of these 170 SNPs in the combined panels have also been published or made publicly available on additional populations and the allele frequencies are stored in the ALFRED database. Noteworthy are consortium datasets and commercial kits providing information for a large subset of the 170 Kidd and Seldin AISNPs. The 26 population samples from the Thousand Genomes consortium (Phase 3)^8^ have complete genotypes and allele frequencies for 169 of the 170 AISNPs. The one SNP (rs10954737) out of 170 not available from the Thousand Genomes dataset is especially helpful in distinguishing Native Americans as a group from other regions of the world and to a lesser extent distinguishing Native American groups from each other. Because of that systematic missing SNP data, we have not included 25 of the 26 Thousand Genomes populations in these analyses (see below).

Here we report selected analyses not previously presented on 81 population samples that now have genotypes and allele frequencies available for 170 markers. This represents the union of SNPs in two of the AISNP panels that are among the few AIM panels that have been studied on a large number of diverse human populations. These consist of the 128 SNPs from the Seldin group^9,10^ and the 55 SNP panel from the Kidd group^11–14^. We also note that a commercial kit, the ThermoFisher Precision ID Ancestry panel, is based on the union of these two panels and includes 165 of the full 170 AISNPs. In recent years an increasing number of studies have also appeared reporting SNP frequencies and/or genotypes for this panel (see e.g. our recent paper^14^ citing six of these studies). Because of these developments we have assembled and analyzed data on all 170 SNPs in 81 distinct population samples. Only populations with data for all 170 SNPs are included here.

## Materials and Methods

### Population samples

Analyses presented include 81 population samples representing the major continental regions of the world that have genotypes available on all 170 of the ancestry informative SNPs in the combined panels from the Kidd Lab (55 SNPs^11^) and the Seldin Lab (128 SNPs^9^). Table S1 lists the populations by geographic region, includes the sample size (N) and the unique sample identifier in the ALFRED database for looking up the description of each sample, and lists the three-character population abbreviations employed in various figures of this report. Some of these 81 population samples have appeared not only in previous publications for both AISNP panels but also in separate studies of specific genes and for studies of haplotypes^15,16^. As documented in previous studies, all samples were collected with informed consent for population genetics studies such as this.

### SNP Genotyping

Table S2 lists the 170 AISNPs including dbSNP rs-numbers, chromosome location, forward strand allele coding, and identifies their membership in the Kidd and/or Seldin panels. The 170 AISNPs were typed at Kidd lab for 76 of the 81 population samples employing TaqMan SNP Genotyping Assays® (Applied Biosystems, Foster City, California, USA) in three microliter reactions following the manufacturer’s instructions. For four of the population samples (Kazakhs, Inner Mongolians, Khamba Tibetans, and BaimaDee) the genotyping was carried out at Dr. Hui Li’s laboratory at Fudan University in Shanghai employing the same TaqMan assays and protocols as used at Yale. For the one population sample (Toscani) from the Thousand Genomes project that was included in the analyses reported here the genotypes for the 169 of 170 AISNPs available were downloaded from the 1000 Genomes Consortium website. The genotyping of the Toscani on the one additional SNP (rs10954737) needed was done at Kidd lab via TaqMan® SNP genotyping assay on the Toscani DNA acquired previously for separate projects^17,18^.

### Statistical analyses

The SNP allele frequencies were obtained by gene counting assuming each locus was a two-allele codominant system. Hardy-Weinberg ratios were tested for all SNP-population combinations. Principal Component Analyses (PCA) used the XLSTAT 2018 software (http://www.xlstat.com/en/about-us/company.html).

We employed version 2.3.4 of the STRUCTURE software^19^ applying the standard admixture model assuming correlated allele frequencies. For the analyses on the full set of 81 populations the program was run 20 times at each K level with 10000 burn-in and 10000 Markov Chain Monte Carlo (MCMC) iterations.

## Results and Discussion

Individuals were omitted if they were missing data for one third or more of the genotypes for any one SNP. The 81 populations analyzed on the combined set of 170 ancestry SNPs, included 3933 individuals. Out of the 668610 possible genotypes (170 SNPs x 3933 individuals) in the dataset there were 12504 or 1.87% missing genotypes. The allele frequencies for the 170 AISNPs in each of the 81 populations are in Table S3. These frequencies are also currently available in the static versions of the ALFRED and FROG-kb databases. Table S4 contains the genotype profiles for the individuals of the 76 populations typed entirely at Kidd Lab. Table S4 also has the genotypes typed at Kidd lab for the Toscani at the one SNP lacking genotypes at the 1000 Genomes Consortium website. There were no significant deviations from Hardy-Weinberg ratios in the context of 81 × 170 = 13770 tests.

**Figure 1:**
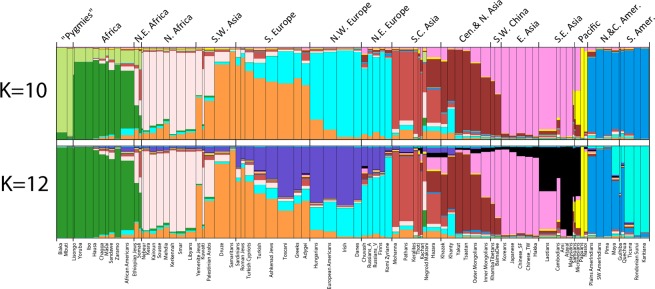
STRUCTURE population bar plots showing estimated cluster membership values for each of 81 populations at K = 10 and K = 12 displaying the highest likelihood run out of 20 runs at each K.

Previous studies of the individual subsets of the full 170 SNPs involved different sets of populations^9–14^ but concluded that the Kidd 55 provided better classification of populations than the Seldin 128. We have now undertaken a proper comparison by conducting STRUCTURE analyses on each SNP panel for exactly the same set of 81 populations. Figure 1 presents population bar charts of the estimated cluster membership values from STRUCTURE runs for all 81 population samples with genotypes available on all 170 AISNPs. The highest likelihood runs out of 20 runs are displayed for K = 10 and K = 12 out of a series of STRUCTURE runs ranging from K = 8 to K = 15. Because the populations in Fig. 1 are represented by width proportional to sample size, the small samples are difficult to distinguish clearly. Thus, Fig. S1 shows the same results but with equal width for each population. Note that several of the populations with the fewest individuals are assigned in part to clusters involving geographically nearby populations, e.g., the Lisongo (LIS) and Samoans (SMO).

The best structure runs for the two subsets, the 55 Kidd and 128 Seldin SNP panels, are in supplemental Figs. S2 and S3. Neither subset has as high an optimal K value as the combined set of 170 SNPs. The additional cluster in the 170 SNP analysis at K = 10 compared to the 55 Kidd SNP result at K = 9 distinguishes three central African (“pygmy”) populations from the other sub-Saharan Africa populations while the cluster patterning in the other world regions appears to be very similar. The best result for the 128 Seldin SNPs is at K = 8. Compared to the results for the other two SNP sets the Seldin panel result does not have clusters distinguishing the South Central Asian populations and the Pacific Island populations but does subdivide the Americas into Northern and Southern clusters.

Since the model used in STRUCTURE assumes that ancestry of individuals comes from one or more of the K distinct “ancestral” sources, logically it follows that a “better” result has a higher percentage of individuals arising primarily from one of the K genetically distinct source populations. Therefore, to quantify the quality of the clusters we have used the level of assignment of individuals to any one of the individual K clusters. We calculated the percentage of individuals that have at least a given percentage of assignment to any cluster. The data are summarized in Table 1. The percentage of individuals that are at least 90% assigned to a cluster is the most telling value. As the number of clusters (K) increases, the percentage of individuals that are partially assigned to multiple clusters increases and the number at the 90% level decreases. This occurs for all three datasets but the numbers are consistently in the following order for each K value: the 170 SNP dataset ranks highest, the Kidd 55 ranks next best, and the Selden 128 ranks lowest. This provides justification for believing the full 170-SNP dataset is better and limiting our further analyses to results for that dataset.

**Table 1:** Comparing cluster membership value estimates (CMVE) of individuals (via STRUCTURE analyses) for different AISNP panels and within panels.

Panel	best *K, other Ks	Count of individuals with CMVE thresholds					Percentage of individuals with CMVE thresholds				
		<60%	≥60%	≥70%	≥80%	≥90%	<60%	≥60%	≥70%	≥80%	≥90%
170 SNPs	K = 8	412	3521	3213	2833	2184	10.5%	89.5%	81.7%	72.0%	55.5%
	K = 9	446	3487	3218	2849	2165	11.3%	88.7%	81.8%	72.4%	55.0%
	*K = 10	502	3431	3153	2755	2035	12.8%	87.2%	80.2%	70.0%	51.7%
	K = 11	646	3287	2949	2543	1809	16.4%	83.6%	75.0%	64.7%	46.0%
	K = 12	729	3204	2844	2388	1656	18.5%	81.5%	72.3%	60.7%	42.1%
55 Kidd SNPs	K = 8	619	3414	3088	2660	1969	15.3%	84.7%	76.6%	66.0%	48.8%
	*K = 9	696	3337	2987	2571	1843	17.3%	82.7%	74.1%	63.7%	45.7%
	K = 10	905	3128	2749	2279	1595	22.4%	77.6%	68.2%	56.5%	39.5%
	K = 11	1088	2945	2550	2074	1387	17.0%	73.0%	63.2%	51.4%	34.4%
	K = 12	1215	2818	2361	1867	1164	30.1%	69.9%	58.5%	46.3%	28.9%
128 Seldin SNPs	*K = 8	762	3164	2795	2346	1633	19.4%	80.6%	71.2%	59.8%	41.6%
	K = 9	1014	2912	2543	2079	1404	25.8%	74.2%	64.8%	53.0%	35.8%
	K = 10	944	2982	2504	2113	1343	24.0%	76.0%	63.8%	53.8%	34.2%
	K = 11	1059	2867	2462	1937	1180	27.0%	73.0%	62.7%	49.3%	30.1%
	K = 12	1174	2752	2367	1881	1150	29.9%	70.1%	60.3%	47.9%	29.3%

The results are for the highest likelihood runs at/near optimal cluster (K) values within each dataset.

There is some variation in the total number of individuals for the 81 populations across the three AISNP sets analyzed because individuals with excessive numbers of missing typings were excluded. An individual was excluded from the analysis of a panel when >33% of SNP typings were missing.

The STRUCTURE results in our earlier report^13^ identified nine optimal clusters in a dataset of 139 population samples analyzing only the 55 Kidd AISNPs. This previous pattern is very similar to the population clustering seen in Fig. 1 for K = 10 based on the full 170 AISNPs. The 81 population samples studied here were all present in that 139 population dataset. At K = 10 the 170-SNP dataset gives a new result compared to earlier analyses. The populations from Sub-Saharan Africa now display partial membership to two different clusters in the current study instead of a single African cluster. Specifically, the two Pygmy populations are in a distinct cluster and that cluster averages 13.9% of the “ancestry” for the other sub-Saharan populations. We note that our African American sample is grouped with the sub-Saharan populations because it shows evidence of only small amounts of European admixture. Other samples of African Americans may well show significantly more admixture. The two Northeast African samples, Ethiopians and Somali, have an intermediate pattern with partial assignment to the two Sub-Saharan clusters and to the North African cluster.

Other than this new “Pygmy” cluster, the other clusters are easy to see in Fig. 1 as distinct colors at K = 10. The interesting aspects are the transitions from a population primarily in one cluster to another population primarily in a different cluster.

**Figure 2:**
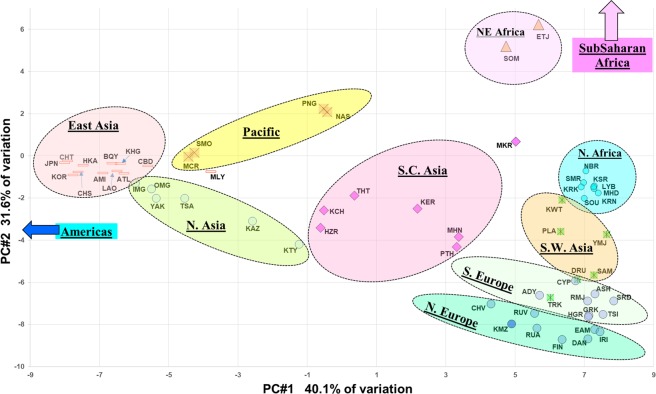
PCA results for 81 populations showing strong clustering by continental regions. Zoom-in view of central clusters (61 of the populations). Population groupings for sub-Saharan Africa (11 populations including Afr-Americans) and Americas (9 populations) are off screen.

The first transition in Fig. 1 is from Sub-Saharan Africa to NE Africa. Both the Ethiopian Jews and the Somali population are small and not sufficiently unique to be clusters on their own. Consequently, they are apportioned by STRUCTURE to genetically “adjacent” populations. In the PCA results (Fig. 2) these two populations are graphically intermediate. Given that this Somali sample comes from workers collected in Pakistan, the partial apportioning to the South Asian cluster is not surprising and may indeed represent some admixture.

The next transitions are from North Africa to Southwest Asia then to Southern Europe and finally to Northern Europe. Three populations show clustering into both the North African and SW Asia population clusters (Yemenite Jews, Kuwaiti, and Palestinians). Then a relatively clean cluster visually (i.e. most individuals have a high membership in the same cluster) appears with the Druze and Samaritans from SW Asia. Sardinians, Roman Jews, and Turkish Cypriots then represent a transition to Southern Europe. The Southern Europeans display partial assignment to the cluster that is essentially a Northern Europe cluster. The Hungarians through the Komi Zyriane form a clear visual cluster for Northern Europe. While the visually clinal pattern from Southwest Asia to Northeast Europe can be seen at K = 10, the clinal pattern is completely obvious at K = 12. When additional Southwest Asia populations have been evaluated, albeit for only the 55 Kidd subset of the 170 SNPs^14^, a more refined and visually clinal picture is seen within SW Asia. Whatever the more detailed pattern of genetic variation in Europe and Southwest Asia, the pattern for these populations with these loci is one of disproportionate assignment of individuals to two (or three) clusters comparing Southwest Asia and Southern Europe with Northwest and Northern Europe summarized in the population averages shown graphically (Figs. S2 and S3). Indeed, a clinal pattern of genetic variation in this region is supported by many other analyses^20,21^.

A clear break in the clustering pattern occurs in Fig. 1 between Northern Europe (the Komi) to South Asia (the Mohanna) at K = 10 and 12. There is only one population sample geographically between these two extremes: the Khanty. The Central Asia cluster is a better fit for the Khanty, but there is some assignment to the European cluster. The South Asian cluster is interrupted by the highly admixed Negroid Makrani. Then, there is a transition to a roughly Central Asian cluster. The Hazara are clearly intermediate with assignment to East Asia and Central Asia in addition to South Asia as has been reported in various studies^22–24^. Many of those populations with strong values for that cluster in the earlier study^13^ could not be included in the current report because genotypes were not available for most of the Seldin Lab AISNPs.

The Central Asian cluster has a transition that is clinal in appearance including the Outer and then Inner Mongolian population samples followed by the Southwestern China populations. Finally, the East Asians through Southeast Asian populations form a very “clean” cluster at K = 10. There is another clinal transition including the Micronesians to the two Melanesian populations that form a clear cluster.

The Native Americans form a quite clean cluster at K = 10. Small amounts of allocation to other clusters amount to little more than noise at this level. Many Native American populations are significantly admixed, probably including some individuals in these population samples. However, when these samples were collected efforts were made to sample individuals that had no known close ancestors of European or African origin.

At K = 12 in Fig. 1 three differences are significant. The “Pygmy” cluster has been incorporated into the large Sub-Saharan Africa cluster. Three additional clusters appear: a cluster (black bars) that includes Southeast Asian populations, another cluster (dark blue) that establishes a clear clinal distribution among various European and S.W. Asian populations, and a division of the Native American samples into two clusters. The North American populations are distinct from the Amazonian populations with the Maya, Guihiba, and Quechua as intermediate. However, the SE Asian cluster along with subclusters for Sub-Saharan Africa and the Americas only occur intermittently among the runs with higher likelihoods of the 20 runs at each of K = 13 to K = 15 in this 170 AISNP dataset. So, while there are indications of additional differentiating information from the 170 AISNP set compared to the 55 Kidd panel, the cluster patterns do not stabilize as higher K levels are explored.

Figure 2 plots PCA results for the 81 populations in a two-dimensional view based on the first two principal components accounting for 71.7% of the variance. Most of the major clusters seen in Fig. 1 are easily discernible from an examination of Fig. 2. The third principal component (not shown) accounts for only an additional 10.1% of the variance and primarily shifts the Native Americans farther from the populations in other world regions. PC#3 also spreads out the East Asian populations relative to each other and moves East Asia a little farther from Europe, SW Asia, North Africa, and the Sub-Saharan region.

### Genetic structure within geographical regions

Additional STRUCTURE analyses were also carried out for subsets of the 81 populations based on various groupings by geographical regions. The 8 principal groupings analyzed include: Sub-Saharan Africa (13 populations), North and Sub-Saharan Africa (21 populations), Europe-Southwest Asia-North Africa (29 populations), Europe-Southwest Asia (21 populations), South Central Asia (7 populations of India, Pakistan), South Central Asia-East and SE Asia-Pacific (28 populations), Core East and SE Asia (18 populations), and Americas (9 populations with and without 3 outlier populations from other regions). The population subsets were explored with the full set of 170 AISNPs to determine whether better differentiation within these regions might be observed without the distraction of genetic diversity from populations outside of those regions. In most regional groupings analyzed no noticeable improvement was observed for the geographical groupings of populations analyzed compared to the clustering of populations observed in Fig. 1 when analyzing all 81 populations together. (See selected images from geographical region analyses in Figs. S4–S7.)

When the Sub-Saharan and Northeast African populations were analyzed as a group, the two Pygmy populations showed a distinct cluster as did the Northeast Africans, the Ethiopians and Somali. There was partial assignment of the three East Africans, the Chagga, Masai, and Sandawe to the Northeast Africa cluster but the Zaramo remained similar to the West Africans.

Analyses of the North Africans, Southwest Asians, and Europeans provided little new except the Samaritans, a population that has undergone considerable genetic drift, separates by K = 4 onto a separate group. By K = 6 the Chuvash and the two Russian groups are primarily assigned to a group that has only partial similarity to other Northern European populations.

In a Structure analysis focused on just the Central, NE, East, and SE Asian populations some more detailed grouping of populations is evident at K = 6 (Fig. S4). The Khanty group forms a clean cluster and the Kazakh show more assignment to that group than to any other. The Yakut and Tsaatan form a group with Outer Mongolians showing considerable assignment to that group. The Inner Mongolians and Southwest China populations form a group, the Lao and Cambodians form a group, and the Taiwan Aboriginal populations, the Ami and Atayal, form a group. The core East Asian populations also form a group. However, many groups are “messy” with lots of different and partial cluster assignments of many individuals.

**Figure 3:**
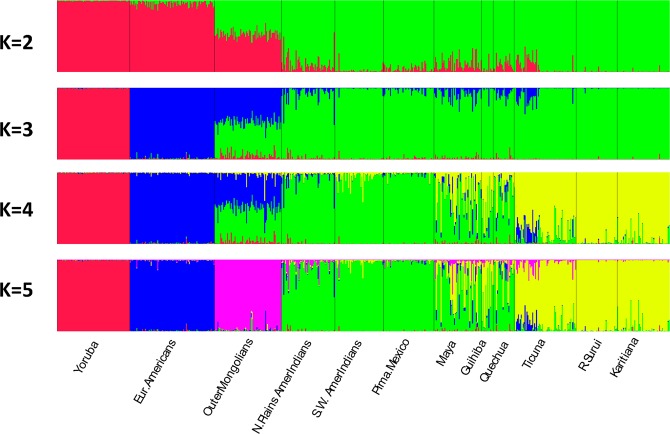
Region—Americas—showing initial differentiation stages into North and South American clusters with low levels of admixture from European and African sources. Individual bar plots from Structure analysis for K = 2 to 5 based on 170 AISNPs. Analysis includes 9 Native American populations and 3 outlier populations (Yoruba, European Americans, and Outer Mongolians). Displaying best of 10 runs at each K.

**Figure 4:**
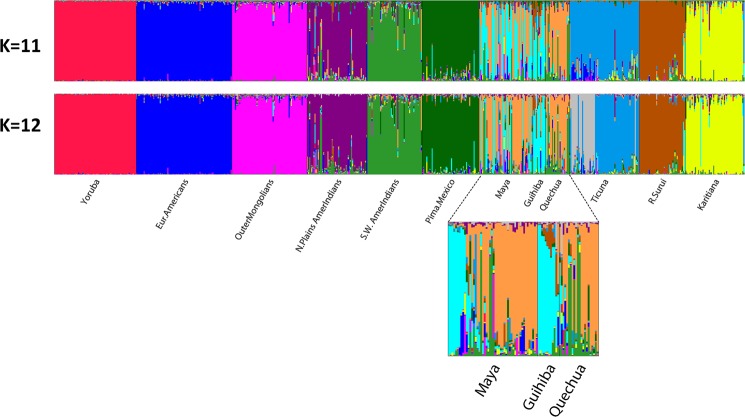
Region—Americas— Individual bar plots from Structure analyses at K = 11 and 12 showing that the differentiation of clusters corresponds increasingly to particular populations at higher K levels for seven of the 9 Native American groups. However, the Maya and Quechua remain more complex with multiple cluster affiliations and it is more difficult to see the predominant light blue cluster for the small number of Guihiba. The extra image inserted below K = 12 displays an expanded view for these three populations with the individuals sorted together that have more similar cluster membership patterns at K = 12. A rather specific gray cluster also appears for about one-third of the Ticuna at K = 12.

In significant contrast to most other regions, considerable differentiation was found among the nine Native American populations when other world regions were excluded. STRUCTURE analyses (not shown) of the nine populations resulted by K = 8 in most populations having assignment to a unique cluster except for the Maya, Guihiba, and Quechua being indistinguishable and the Ticuna subdividing into two clusters. All of the clusters showed noticeable partial assignment to other clusters. STRUCTURE analyses were then performed with three outlier groups and the nine Native American populations. Three outliers–Yoruba, European Americans, and Outer Mongolians–were chosen to help evaluate both the extent to which admixture from West African and European populations in recent generations contributes to the results as well as how the Mongolians would affect clustering. Results were generated for K = 2 to 13 with 20 runs at each K level. Selected runs are illustrated in Figs. 3 and 4 (Fig. 3 displays K = 2 to 5; Fig. 4 shows K = 11 and 12). At K = 2 through K = 4 the intermediate position of the Mongolians is evident. At K = 4 the Native Americans divide into a North American cluster (related to the Mongolians) and an Amazonian American cluster with the Maya, Guihiba, and Quechua as an intermediate cluster. The outliers separated from each other and the Native Americans at K = 5 and remained separate as the Native Americans continued to show increasing levels of distinction. By K = 9 each population constituted its own cluster except for the Maya, Guihiba, and Quechua. At K = 11 the Guihiba separates from the Maya and Quechua. At K = 12 the Ticuna sample divides into two groups, apparently a side effect of the use of mtDNA to establish two sets of cell lines from a larger collection of Ticuna samples. At higher K values no new clusters appeared and the existing clusters became less clearly distinct. The inclusion of the Yoruba and European American outliers indicates low levels of admixture for these biogeographic areas in these Native American groups: from Europeans (0–6%) and to a lesser degree from Africans (0–1%) depending on individual population and K level examined.

In general, the results with the three outlier populations present were very similar to those found when the nine Native American populations were analyzed without the Mongolian outlier. The inclusion of the Yoruba and European American outliers shows low levels of allocation to these biogeographic areas in the Native American groups: from Europeans (0–6%) and to a lesser degree from Africans (0–1%) depending on individual population and K level examined.

## Conclusions

Our study illustrates both the value of extensive SNP data on a large number of populations and the difficulty in assembling such a dataset involving a large number of populations and individuals genotyped for the same set of SNPs. In order to add more populations we would have had to use fewer loci. We chose to opt for more comparably typed loci at this time and excluded the 1000 Genomes populations even though the missing SNP in the 1000 Genomes Project will make little if any difference in analyses. We have seen small and intermediate populations show partial assignment to two or more clusters. Usually in the literature this is often considered an indication of admixture but our analyses have shown that it is most conservative, barring historical evidence to the contrary, to consider these populations as simply intermediate for whatever reason. The dependence of the clustering pattern on the selection of populations is shown by the assignment of essentially all individuals in the Outer Mongolian sample to two groups, Europeans and Native Americans, until a sufficient number of clusters are allowed for the Outer Mongolians to be assigned to their own cluster. Similarly, in other analyses of regional subsets of populations, populations with partial assignment to two clusters become a clear cluster. We note the Northeast African populations as another example.

There are biomedical implications from our findings. For example, the North African populations analyzed are clearly genetically distinguishable from most Middle East (Southwest Asian) populations and from Europeans. Northern Europeans are genetically distinguishable from Southern Europeans. Perhaps the most significant result is that “Asians”, as used in much biomedical literature, comprises four clearly distinguishable groups–South Asians, Central Asians, East Asians, and Southeast Asians–with many populations intermediate among the more extreme of those four clusters. It would not be surprising if even more discernible population groupings in Asia and elsewhere will emerge as the population sampling of human genetic diversity continues to improve and better AISNP panels are developed.

## Supplementary information

Supplementary Information

Supplementary Dataset

## Data Availability

The allele frequencies for all 170 AISNPs have been entered into the databases–ALFRED and FROG-kb–for the populations included in this report. They can also be found in Table S3. The individual genotype profiles for the 170 AISNPs in each the 76 populations typed at Kidd lab are available in Table S4 along with the typings on the Toscani for the one SNP out of the 170 AISNPs not currently available at the Thousand Genomes website.
