# Supplementary material for: Population relationships based on 170 ancestry SNPs from the combined Kidd and Seldin panels
Source: Sci Rep. 2019 Dec 11;9:18874. doi: 10.1038/s41598-019-55175-x (PMC6906462; doi:10.1038/s41598-019-55175-x)

Supplementary Material:
Tables S1 and S2

Submitted to *Scientific Reports*

"Population relationships based on 170 ancestry SNPs from the combined Kidd and Seldin panels"

A.J. Pakstis[1], W.C. Speed[1], U. Soundararajan[1], H. Rajeevan[2], J.R. Kidd[1], H. Li[3], K.K. Kidd[1]

[1] Department of Genetics, Yale University School of Medicine, New Haven, CT 06520 USA

[2] Center for Medical Informatics, Yale University School of Medicine, New Haven, CT 06520 USA

[3] M.O.E. Key Laboratory of Contemporary Anthropology, School of Life Sciences, Fudan University, Shanghai 200433, China

Corresponding Author: Kenneth.Kidd@yale.edu

Table S1. The 81 populations organized by geographical location.

World Region	Population	ALFRED Sample UID	N	Abbrev.
Africa, Central	Biaka, Central African Rep.	SA000005F	69	BIA
	Mbuti, Ituri Forest, D.R. Congo	SA000006G	39	MBU
	Lisongo	SA002770Q	8	LIS
Africa, West	Yoruba, Benin City, Nigeria	SA000036J	77	YOR
	Ibo, Nigeria	SA000099S	48	IBO
	Hausa, Nigeria	SA000100B	39	HSA
Africa, East	Chagga, Tanzania	SA000099S	45	CGA
	Masai, Tanzania	SA000854R	20	MAS
	Sandawe, Tanzania	SA001773S	40	SND
	Zaramo, Tanzania	SA002586V	40	ZRM
	African Americans	SA000101C	89	AAM
Africa, N.E.	Ethiopian Jews	SA000015G	32	ETJ
	Somali, collected in Pakistan	SA002138O	17	SOM
Africa, North	Nebeur, Tunisia	SA004254P	11	NBR
	Kesra, Tunisia	SA004255Q	43	KSR
	Kairoun, Tunisia	SA004256R	45	KRN
	Sousse, Tunisia	SA004257S	46	SOU
	Mehdia, Tunisia	SA004258T	40	MHD
	Kerkennah, Tunisia	SA004259U	46	KRK
	Smar, Tunisia	SA004260M	61	SMR
	Libyans; from 6 locations in Libya	SA004261N	69	LYB
Asia, South West	Yemenite Jews	SA000016H	42	YMJ
	Kuwaiti	SA002765U	14	KWT
	Palestinian Arabs	SA002766V	68	PLA
	Druze, Israel	SA000047L	102	DRU
	Samaritans, Israel	SA000098R	39	SAM
Europe, South	Sardinians	SA002768X	34	SRD
	Roman Jews, Italy	SA000096P	27	RMJ
	Turkish Cypriots	SA004333N	59	CYP
	Turkish, Istanbul, Turkey	SA004310I	80	TRK
	Ashkenazi Jews	SA000490N	79	ASH
	Toscani, Italy	SA004057Q	107	TSI
	Greeks,Thesaloniki, Greece	SA002767W	52	GRK
	Adygei	SA000017I	54	ADY
	Hungarians	SA002023H	89	HGR
Europe, North	European Americans	SA000020C	89	EAM
	Irish	SA000057M	114	IRI
	Danes	SA000007H	51	DAN
	Chuvash	SA000491O	42	CHV

	Russians, Archangelsk	SA001530J	33	RUA
	Russians, Vologda	SA000019K	47	RUV
	Finns	SA000018J	34	FIN
Siberia, West	Komi Zyriane	SA000489V	47	KMZ
Asia, So. Central	Mohanna, Pakistan	SA002139P	48	MHN
	Pathans, Pakistan	SA002873U	95	PTH
	Keralites, India	SA001854S	30	KER
	Thoti, India	SA000077O	14	THT
	Kachari, Assam state, India	SA000040E	17	KCH
	Negroid Makrani, Pakistan	SA002137N	26	MKR
	Hazara, Pakistan	SA002140H	95	HZR
Asia, Central	Khazak, Xinjiang, China	SA002429R	44	KAZ
Siberia	Khanty	SA000488U	50	KTY
	Yakut	SA000011C	51	YAK
Asia, NorthEast	Tsaatan	SA004036N	51	TSA
	Outer Mongolians	SA004035M	70	OMG
	Inner Mongolians, China	SA002431K	64	IMG
(SW China)	Khamba Tibetans, Sichuan, China	SA002434N	29	KHG
(SW China)	Baima Dee, Sichuan, China	SA002432L	41	BQY
Asia, East	Koreans	SA000936S	54	KOR
	Japanese	SA000010B	48	JPN
	Chinese, San Francisco, USA	SA000009J	58	CHS
	Chinese, Taiwan	SA000001B	50	CHT
	Hakka, Taiwan	SA000003D	41	HKA
	Laotians	SA001853R	118	LAO
	Cambodians	SA000022E	24	CBD
	Ami, Taiwan	SA000002C	40	AMI
	Atayal, Taiwan	SA000021D	42	ATL
	Malaysians	SA000097Q	11	MLY
Pacific	Samoans	SA000072J	9	SMO
	Micronesians	SA000063J	34	MCR
	Papuans, New Guinea	SA000084M	22	PNG
	Nasioi, Bougainville, Solomon Islands	SA000012D	23	NAS
America, North	Plains AmerIndians	SA000023F	56	NPA
	Southwest AmerIndians	SA000025H	51	SWA
	Pima, northern Mexico	SA000026I	53	PMX
	Maya, Yucatan, Mexico	SA000013E	50	MAY
America, South	Guihiba speakers, Colombia	SA000055K	12	GHB
	Quechua, Peru	SA000069P	22	QUE
	Ticuna, Amazon region, Brazil	SA000027J	65	TIC
	Rondonian Surui, Amazon region, Brazil	SA000014F	43	SUR
	Karitiana, Amazon region, Brazil	SA000028K	55	KAR

Table S2. The 170 autosomal AISNPs in the combined Kidd and Seldin panels sorted by rs-number.

	AISNP Panel Membership	Chr	Nucleotide position GRCh37.p13	Foot-Note	dbSNP rs-number	Forward Strand Alleles
1	Seldin 128	4	41,554,364		rs10007810	AG
2	Seldin 128	8	4,190,793		rs10108270	AC
3	Seldin 128	7	139,447,377		rs10236187	AC
4	Seldin 128	6	4,747,159		rs1040045	AG
5	Seldin 128	1	168,159,890		rs1040404	AG
6	Seldin 128	2	145,769,943		rs10496971	GT
7	Kidd 55	2	158,667,217		rs10497191	CT
8	Seldin 128	3	2,208,832	1	rs10510228	AG
9	Seldin 128	9	28,628,500		rs10511828	CT
10	Seldin 128	17	69,512,099		rs10512572	AG
11	Seldin 128	9	120,130,206		rs10513300	CT
12	Kidd 55	11	113,296,286		rs1079597	CT
13	Seldin 128	11	7,850,316		rs10839880	CT
14	Seldin 128	7	83,533,047	2	rs10954737	CT
15	Seldin 128	11	66,898,492		rs11227699	AG
16	Kidd&Seldin	17	62,987,151		rs11652805	CT
17	Seldin 128	1	55,663,372		rs12130799	AG
18	Kidd 55	4	100,239,319		rs1229984	CT
19	Kidd&Seldin	15	36,220,035		rs12439433	AG
20	Kidd 55	3	121,459,589		rs12498138	AG
21	Seldin 128	8	86,424,616		rs12544346	AG
22	Seldin 128	3	120,522,716		rs12629908	AG
23	Seldin 128	5	79,085,726		rs12657828	AG
24	Kidd 55	15	28,365,618		rs12913832	AG
25	Seldin 128	22	18,076,546		rs1296819	AC
26	Seldin 128	1	42,360,270		rs1325502	AG
27	Seldin 128	2	79,864,923		rs13400937	GT
28	Seldin 128	4	73,245,191		rs1369093	CT
29	Seldin 128	1	186,149,032		rs1407434	AG
30	Seldin 128	9	12,672,320	1	rs1408801	AG
31	Kidd 55	15	48,426,484		rs1426654	AG
32	Kidd 55	8	31,896,592		rs1462906	CT
33	Seldin 128	8	28,941,305		rs1471939	CT
34	Seldin 128	5	165,739,982		rs1500127	CT
35	Seldin 128	12	118,889,488		rs1503767	GT
36	Seldin 128	12	17,407,792		rs1513056	AG
37	Seldin 128	3	188,574,996		rs1513181	AG
38	Seldin 128	2	201,021,954		rs1569175	CT
39	Kidd 55	13	41,715,282		rs1572018	CT

40	Kidd 55	5	33,951,693		rs16891982	CG
41	Kidd 55	11	61,597,212		rs174570	CT
42	Seldin 128	14	20,818,131		rs1760921	CT
43	Kidd 55	17	48,726,132		rs17642714	AT
44	Kidd 55	15	28,197,037		rs1800414	CT
45	Kidd 55	2	17,901,485		rs1834619	AG
46	Seldin 128	11	15,838,137		rs1837606	CT
47	Seldin 128	6	168,665,760		rs1871428	AG
48	Kidd 55	8	145,639,681		rs1871534	CG
49	Kidd 55	2	17,362,568		rs1876482	AG
50	Seldin 128	17	1,401,613		rs1879488	AC
51	Kidd&Seldin	6	90,518,278		rs192655	AG
52	Seldin 128	14	58,238,687		rs1950993	GT
53	Seldin 128	8	140,241,181		rs2001907	CT
54	Kidd&Seldin	14	99,375,321		rs200354	GT
55	Kidd 55	22	41,697,338		rs2024566	AG
56	Seldin 128	3	179,964,727		rs2030763	AG
57	Seldin 128	17	53,788,280		rs2033111	AG
58	Kidd 55	18	35,277,622		rs2042762	CT
59	Seldin 128	12	109,277,720		rs2070586	AG
60	Seldin 128	9	135,933,122		rs2073821	CT
61	Seldin 128	17	73,782,191		rs2125345	CT
62	Seldin 128	12	47,676,950		rs214678	CT
63	Kidd 55	13	42,579,985		rs2166624	AG
64	Kidd 55	8	122,124,302		rs2196051	AG
65	Kidd 55	12	112,211,833		rs2238151	CT
66	Seldin 128	16	19,272,908	1	rs2269793	GT
67	Seldin 128	9	93,641,199		rs2306040	CT
68	Seldin 128	7	42,380,071		rs2330442	AG
69	Seldin 128	14	52,607,967		rs2357442	AC
70	Seldin 128	6	51,611,470	1	rs2397060	CT
71	Seldin 128	12	11,701,488		rs2416791	AG
72	Seldin 128	6	12,535,111		rs2504853	CT
73	Seldin 128	19	55,614,923		rs2532060	CT
74	Kidd 55	17	41,056,245		rs2593595	AG
75	Kidd&Seldin	2	109,579,738		rs260690	AC
76	Seldin 128	2	179,606,538		rs2627037	AG
77	Seldin 128	4	179,399,523		rs2702414	AG
78	Kidd 55	1	159,174,683		rs2814778	CT
79	Seldin 128	21	37,885,625		rs2835370	CT
80	Seldin 128	15	74,734,500		rs2899826	AG
81	Seldin 128	11	24,010,530		rs2946788	GT
82	Seldin 128	16	85,183,682		rs2966849	AG
83	Seldin 128	1	6,550,376		rs2986742	CT
84	Kidd 55	20	62,159,504		rs310644	CT

85	Seldin 128	1	68,849,687		rs3118378	AG
86	Seldin 128	5	2,364,626		rs316598	CT
87	Seldin 128	1	242,342,504		rs316873	CT
88	Seldin 128	7	32,179,124		rs32314	CT
89	Seldin 128	5	35,037,115		rs37369	CT
90	Kidd&Seldin	1	101,709,563		rs3737576	CT
91	Seldin 128	19	52,901,905		rs3745099	AG
92	Seldin 128	14	105,679,055		rs3784230	AG
93	Seldin 128	9	71,659,280		rs3793451	CT
94	Seldin 128	10	50,841,704		rs3793791	CT
95	Kidd 55	4	100,244,319		rs3811801	AG
96	Kidd 55	9	127,267,689		rs3814134	AG
97	Kidd 55	6	136,482,727		rs3823159	AG
98	Kidd 55	2	109,513,601		rs3827760	AG
99	Seldin 128	4	85,309,078		rs385194	AG
100	Seldin 128	20	54,000,914		rs3907047	CT
101	Kidd 55	18	67,578,931		rs3916235	CT
102	Seldin 128	8	13,359,500		rs3943253	AG
103	Kidd 55	17	40,658,533		rs4411548	CT
104	Seldin 128	6	163,221,792		rs4458655	CT
105	Seldin 128	6	145,055,331		rs4463276	AG
106	Kidd 55	17	53,568,884		rs4471745	AG
107	Kidd 55	16	89,730,827		rs459920	CT
108	Seldin 128	2	29,538,411		rs4666200	AG
109	Seldin 128	2	37,941,396		rs4670767	GT
110	Seldin 128	7	73,454,199		rs4717865	AG
111	Seldin 128	10	75,300,994		rs4746136	AG
112	Seldin 128	16	10,975,311		rs4781011	GT
113	Seldin 128	18	9,420,504		rs4798812	AG
114	Seldin 128	18	19,651,982	1	rs4800105	CT
115	Seldin 128	22	32,366,359		rs4821004	CT
116	Kidd 55	4	38,815,502		rs4833103	AC
117	Seldin 128	10	134,650,103		rs4880436	CT
118	Kidd&Seldin	18	67,867,663		rs4891825	AG
119	Seldin 128	1	27,931,698		rs4908343	AG
120	Kidd 55	10	94,921,065		rs4918664	AG
121	Seldin 128	10	115,316,812		rs4918842	CT
122	Seldin 128	1	212,786,883		rs4951629	CT
123	Seldin 128	3	30,415,612		rs4955316	GT
124	Seldin 128	16	740,466		rs4984913	AG
125	Seldin 128	22	48,207,872		rs5768007	CT
126	Seldin 128	20	10,195,433		rs6104567	GT
127	Seldin 128	5	177,863,083		rs6422347	CT
128	Seldin 128	5	43,711,378		rs6451722	AG
129	Seldin 128	7	151,873,853		rs6464211	CT

130	Seldin 128	1	18,170,886		rs647325	AG
131	Seldin 128	1	12,608,178		rs6541030	AG
132	Seldin 128	3	79,399,575		rs6548616	CT
133	Seldin 128	5	155,471,714		rs6556352	CT
134	Kidd 55	12	112,241,766		rs671	AG
135	Kidd 55	2	136,707,982		rs6754311	CT
136	Kidd 55	8	110,602,317		rs6990312	GT
137	Seldin 128	7	97,695,363		rs705308	AC
138	Kidd 55	18	40,488,279		rs7226659	GT
139	Seldin 128	18	49,781,544		rs7238445	AG
140	Kidd 55	19	4,077,096		rs7251928	AC
141	Seldin 128	7	12,669,251		rs731257	AG
142	Kidd 55	13	49,070,512		rs7326934	CG
143	Seldin 128	3	147,750,355		rs734873	AG
144	Kidd 55	15	45,152,371		rs735480	CT
145	Seldin 128	2	14,756,349		rs7421394	AG
146	Kidd&Seldin	1	151,122,489		rs7554936	CT
147	Kidd&Seldin	4	105,375,423		rs7657799	GT
148	Kidd 55	5	170,202,984		rs7722456	CT
149	Seldin 128	12	56,163,734		rs772262	AG
150	Seldin 128	6	21,911,616		rs7745461	AG
151	Seldin 128	7	130,742,066		rs7803075	AG
152	Seldin 128	8	122,908,503		rs7844723	CT
153	Kidd&Seldin	2	7,968,275		rs798443	AG
154	Kidd&Seldin	13	34,847,737		rs7997709	CT
155	Seldin 128	14	67,886,781		rs8021730	GT
156	Seldin 128	15	92,105,708		rs8035124	AC
157	Seldin 128	19	33,652,247		rs8113143	AC
158	Seldin 128	16	65,406,708		rs818386	CT
159	Kidd&Seldin	5	6,845,035		rs870347	AC
160	Seldin 128	18	75,056,284		rs874299	CT
161	Seldin 128	18	59,333,108		rs881728	AC
162	Kidd 55	7	28,172,586		rs917115	CT
163	Seldin 128	4	5,390,637		rs9291090	AC
164	Seldin 128	13	27,624,356		rs9319336	CT
165	Seldin 128	14	83,472,868		rs946918	GT
166	Seldin 128	11	120,644,447		rs948028	AC
167	Kidd&Seldin	13	111,827,167		rs9522149	CT
168	Seldin 128	13	75,993,887		rs9530435	CT
169	Seldin 128	3	39,146,429		rs9809104	CT
170	Seldin 128	3	135,914,476		rs9845457	AG

Footnotes

1. Five SNPs that are not included in the ThermoFisher Precision ID ancestry panel.
2. SNP (rs10954737) lacking typings at the Thousand Genomes Consortium website (Phase 3)

Supplementary Material:
Figures S1 to S7

Submitted to *Scientific Reports*

"Population relationships based on 170 ancestry SNPs from the combined Kidd and Seldin panels"

A.J. Pakstis[1], W.C. Speed[1], U. Soundararajan[1], H. Rajeevan[2], J.R. Kidd[1], H. Li[3], K.K. Kidd[1]

[1] Department of Genetics, Yale University School of Medicine, New Haven, CT 06520 USA
[2] Center for Medical Informatics, Yale University School of Medicine, New Haven, CT 06520 USA
[3] M.O.E. Key Laboratory of Contemporary Anthropology, School of Life Sciences, Fudan University, Shanghai 200433, China

Corresponding Author: Kenneth.Kidd@yale.edu

Figure S1. **Alternate version of Figure 1 components. Population bar plots from STRUCTURE results at K= 10 and 12. The width of each bar the same. The population order is the same here as in Figure 1.**

K=10

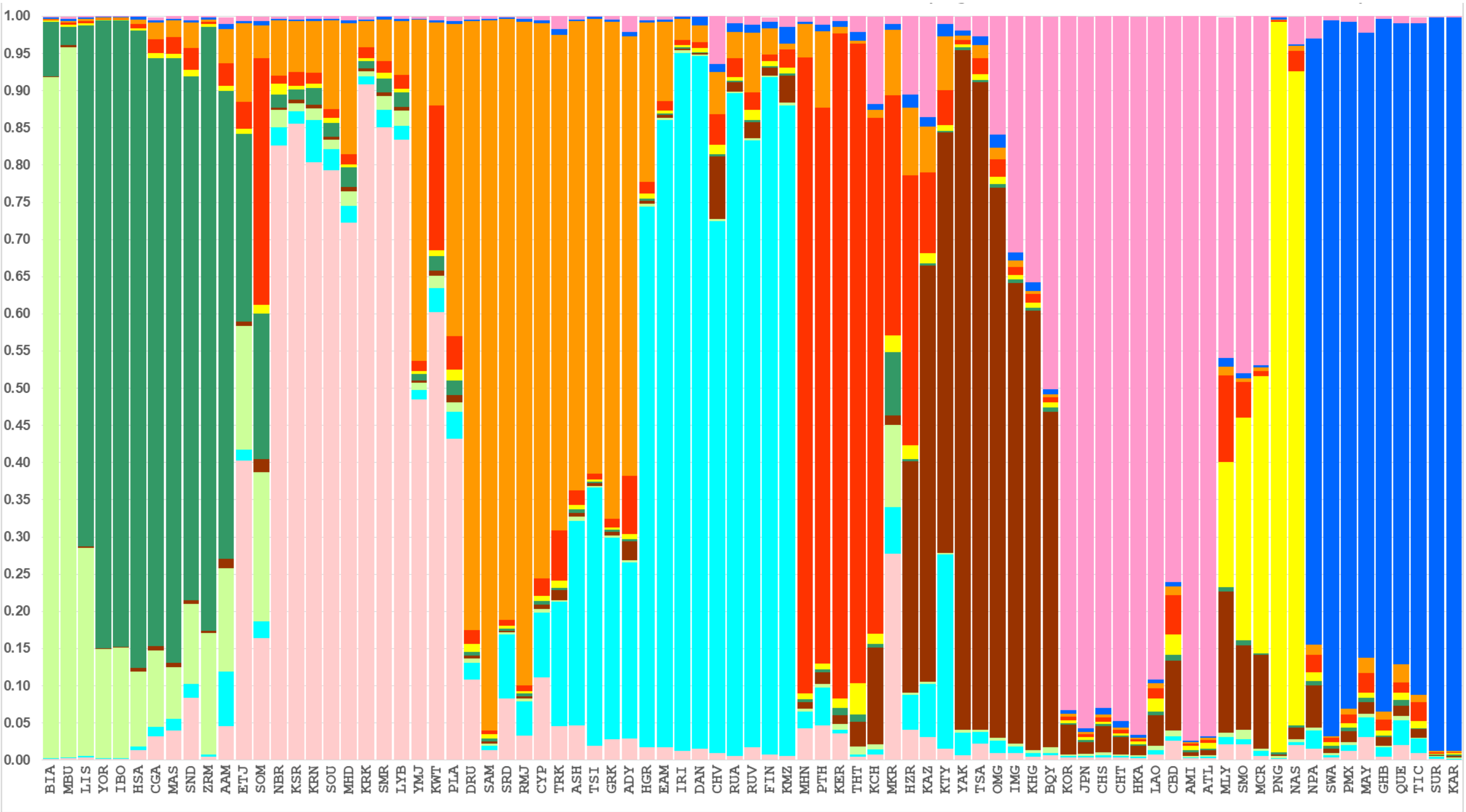

K=12

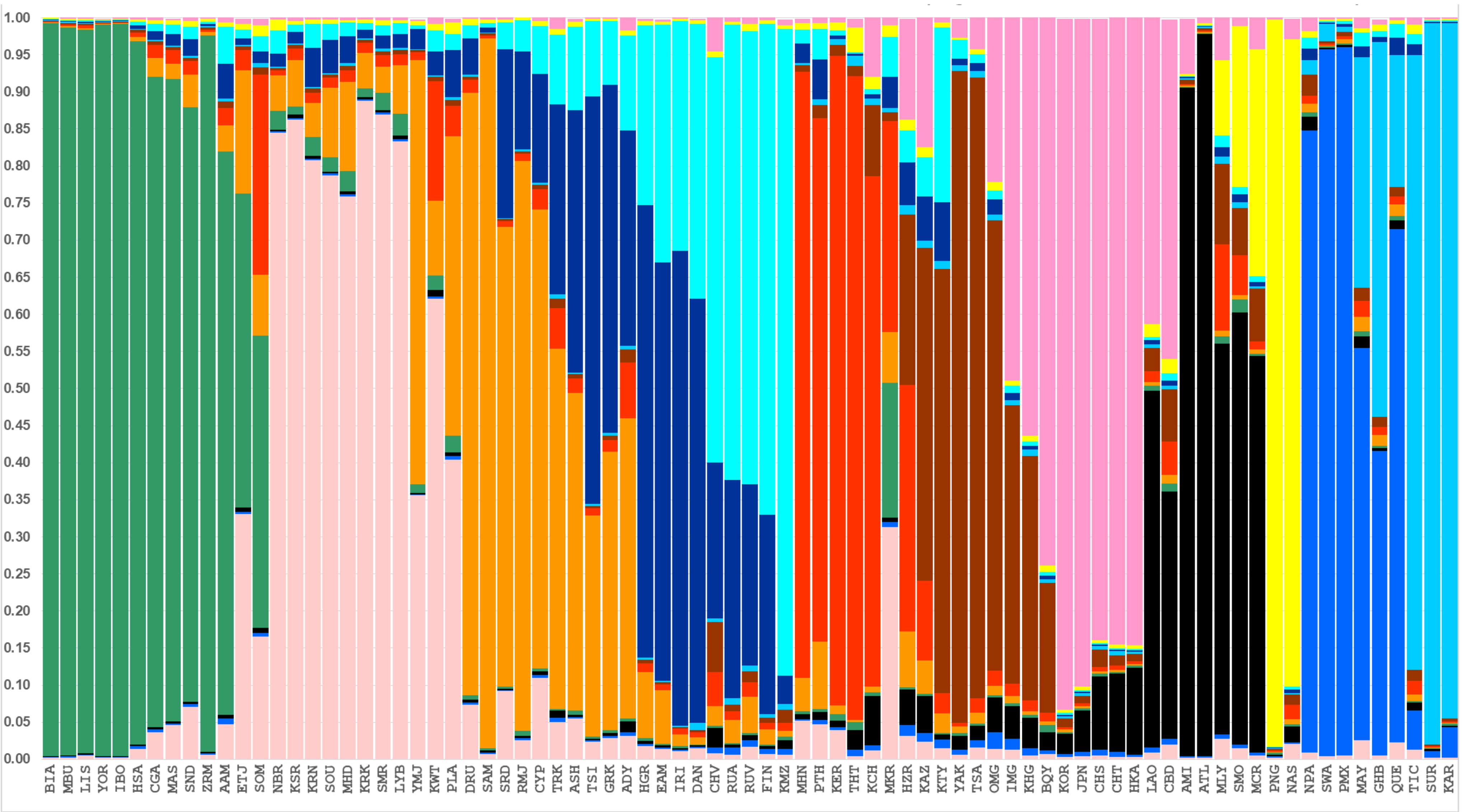
1.00
0.95
0.90
0.85
0.80
0.75
0.70
0.65
0.60
0.55
0.50
0.45
0.40
0.35
0.30
0.25
0.20
0.15
0.10
0.05
0.00
BIA MBU LIS YOR IBO HSA CGA MAS SND ZRM AAM ETJ SOM NBR KSR KRN SOU MHD KRK SMR LYB YMJ KWT PLA DRU SAM SRD RMJ CYP TRK ASH TSI GRK ADY HGR EAM IRI DAN CHV RUA RUV FIN KMZ MHN PTH KER THT KCH MKR HZR KAZ KTY YAK TSA OMG IMG KHG BQY KOR JPN CHS CHT HKA LAO CBD AMI ATL MLY SMO MCR PNG NAS NPA SWA PMX MAY GHB QUE TIC SUR KAR

Figure S2. **STRUCTURE population bar plots showing estimated cluster membership values of 81populations for 55 Kidd SNPs and for 128 Seldin SNPs. Optimal K values are at K=9 for the 55 Kidd SNPs and at K=8 for the 128 Seldin SNPs**

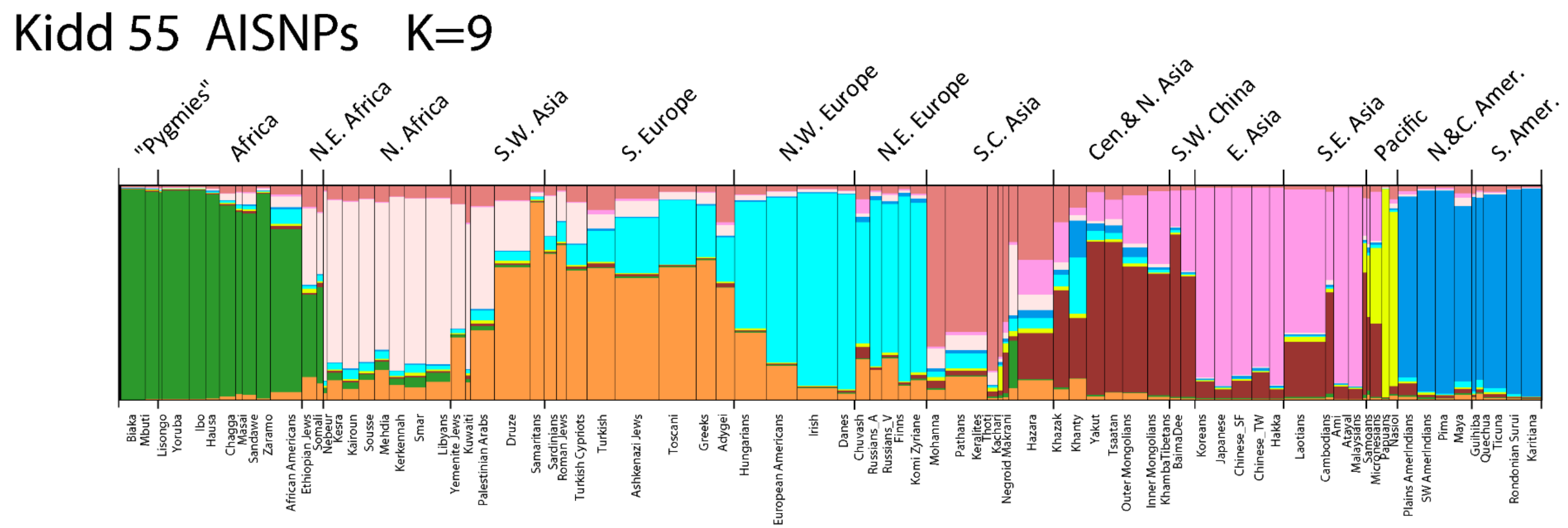

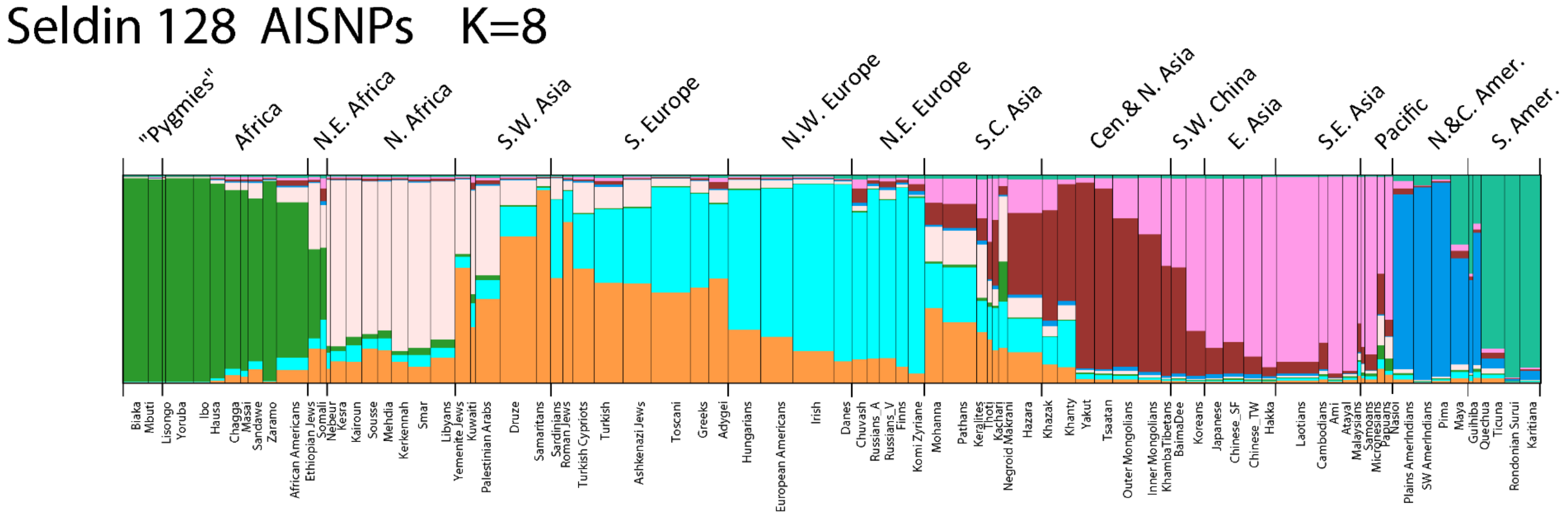

Figure S3. **Alternate version of Figure S2 components with the width of each bar the same. STRUCTURE population bar plots showing estimated cluster membership values of 81 populations for 55 Kidd SNPs and for 128 Seldin SNPs. Optimal K values are at K=9 for the 55 Kidd SNPs and at K=8 for the 128 Seldin SNPs.**

Kidd 55 AISNPs K=9

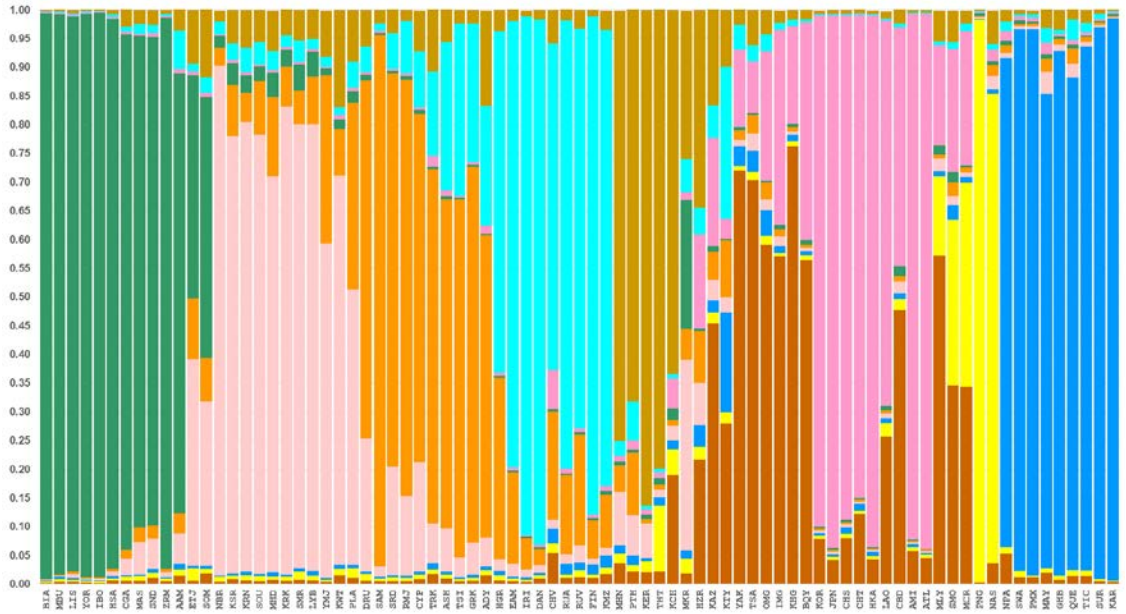

Seldin 128 AISNPs K=8

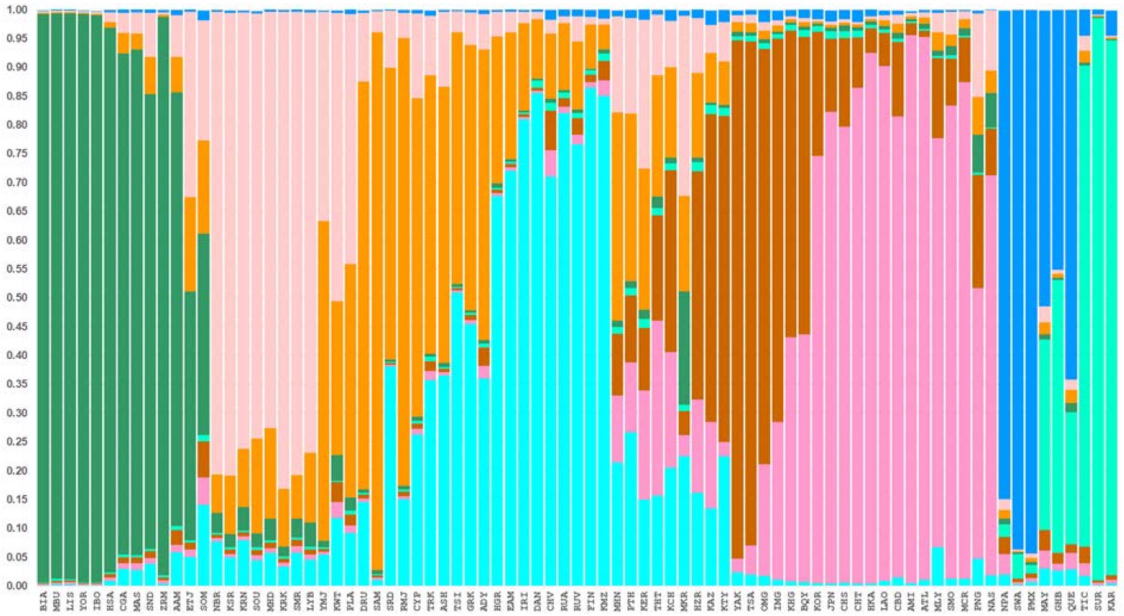

STRUCTURE software individual bar plots--selected geographical region analysis results.

--Displaying the highest likelihood result at the various K levels shown.

--See supplementary Table S1 for full names (along with other information on the groups analyzed) for the 3-character population abbreviations used in the images below.

Suppl. Figure S4. Core East Asia; K=6; 18 populations

Suppl. Figure S5. South Central Asia; K=3; 7 populations

Suppl. Figure S6. Sub-Saharan Africa; K=3; 13 populations

Suppl. Figure S7. North Africa, SW Asia, Europe; K=3 and K=5; 29 populations

Suppl. Figure S4

“Core East Asia”—Siberia and Cen. Asia, N.E. Asia, major East Asia, S.E. Asia—18 populations

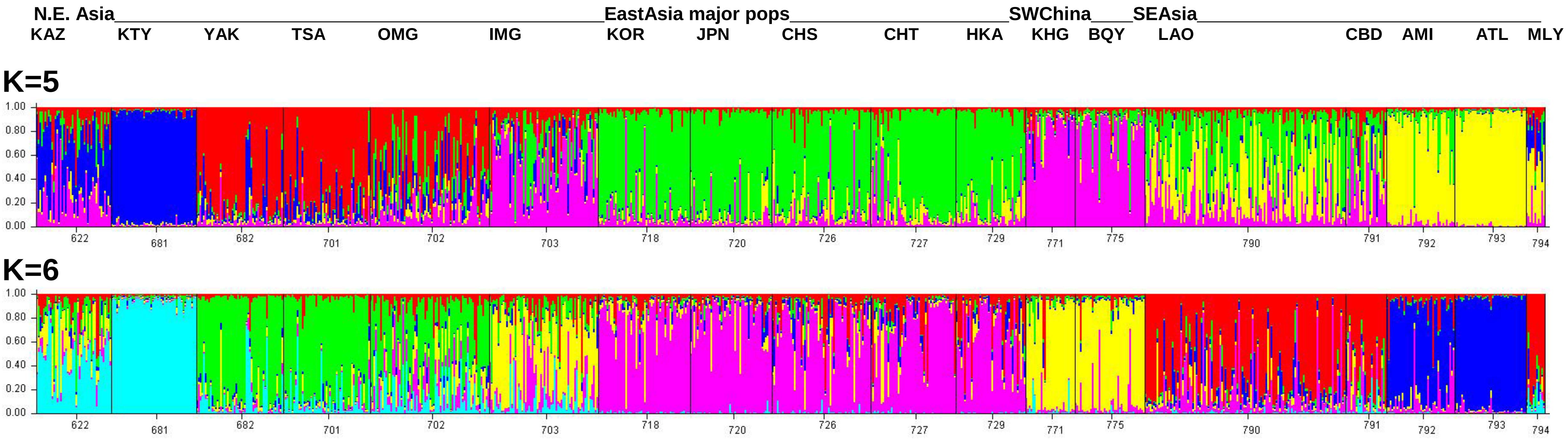

Suppl. Figure S5

“South Central Asia” –7 pops (India, Pakistan populations, plus Kazakhs as outlier)

>> At K=2 we see a result that has occurred before with the 55 Kidd AISNPs in different analyses that include populations from many world regions. The Hazara, Kazakhs and Kachari separate from the Mohanna/Pathans/Keralites/Thoti.

>> <u>At K=3 we do see an interesting minor result that we have not noticed before.</u> The Thoti and Keralites differentiate somewhat from the Mohanna and Pathans as well as the Hazara and Kazakhs.

<u>Note:</u> The Negroid Makrani—sampled in Pakistan--were not included in this regional analysis due to their diverse ancestry.

K=2

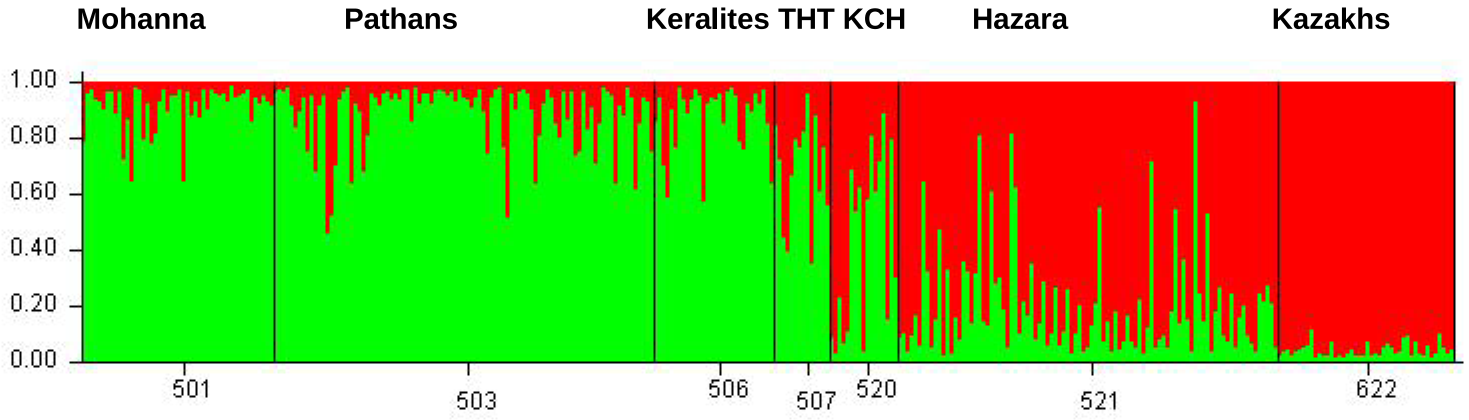

K=3

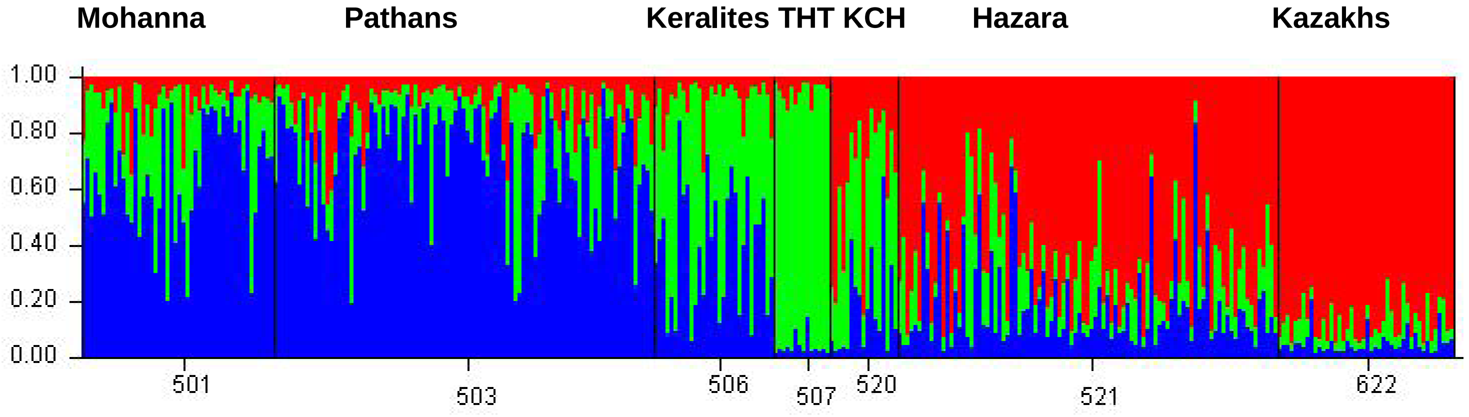

Suppl. Figure S6
“Sub-Saharan Africa”---13 populations

>> At K=3 we see results that have appeared before in a number of variations in global STRUCTURE analyses based on the 55 Kidd AISNPs and also with microhaplotypes. Here the Biaka and Mbuti strongly differ from West and East African populations. Sometimes the Lisongo cluster more strongly with the Biaka and Mbuti.
The West African Yoruba, Ibo, and Hausa along with the Zaramo from East Africa have green cluster bars predominantly. The red cluster bars characterize the Ethiopians and the Somali here while we see more intermediate patterns for other East Africans such as the Chagga, Masai, and Sandawe groups.
The African Americans (AAM) interestingly and typically resemble the mixed cluster patterns of various East African groups. We know that the AAM have predominantly West African ancestry along with some European ancestry and a small amount of Native American ancestry. In the context of the particular SNPs and populations analyzed the STRUCTURE analysis results in their cluster pattern resembling East African populations.

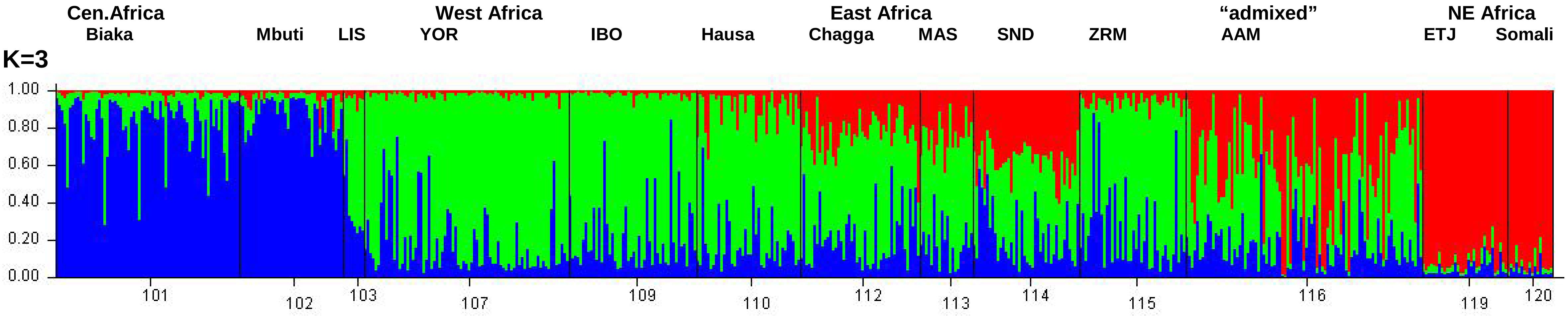

Suppl. Figure S7

“North Africa, SW Asia, Europe”---29 populations

>>Cluster patterns below are pattern variations previously seen in STRUCTURE analyses based on the 55 Kidd lab ancestry informative SNPs and populations from around the world. From South to North approximately in SW Asia--Europe we see a clinal change in the cluster patterns. The strong single cluster estimates for the Samaritans (SAM) occurs frequently in various SNP datasets we have tested. At K=5 the predominant light blue cluster seen in the Chuvash and the two Russian population samples and to a lesser extent in the Finns has appeared for the 55 Kidd AISNPs when analyzing datasets encompassing populations from around the world; however, this regional pattern has usually appeared in those earlier analyses at higher K values and less strongly in conjunction with cluster membership patterns in other world regions that are not optimal (i.e. complex multi-cluster assignments of individuals).

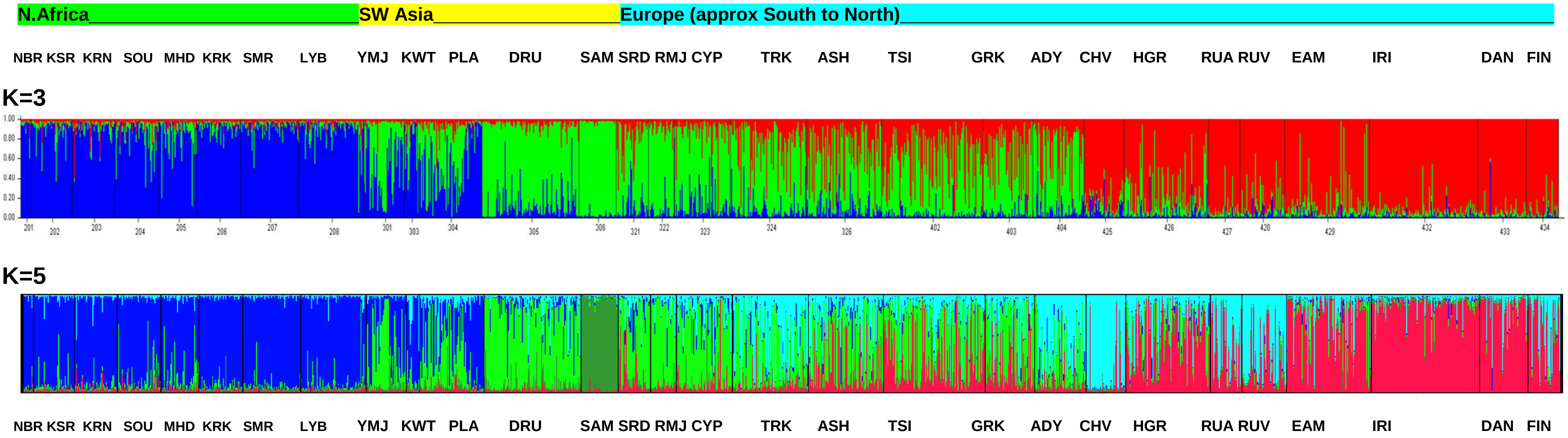

Supplement: Supplementary file 1 — Supplementary Information [file 41598_2019_55175_MOESM1_ESM.pdf]
